# Successful treatment of hairy cell leukemia variant with obinutuzumab

**DOI:** 10.1007/s00277-021-04559-z

**Published:** 2021-06-04

**Authors:** Diana Al-Sarayfi, Freek O. Meeuwes, Thijs Oude Munnink, Wouter Plattel, Stefano Rosati, Estella Matutes, Marcel Nijland

**Affiliations:** 1 Department of Hematology, University Medical Center Groningen, Hanzeplein 1, 9700 RB Groningen, The Netherlands; 2 Department of Hematology, Martini Hospital, Groningen, The Netherlands; 3 Department of Clinical Pharmacy and Pharmacology, University Medical Center Groningen, Hanzeplein 1, 9700 RB Groningen, The Netherlands; 4 Department of Pathology, University Medical Center Groningen, Hanzeplein 1, 9700 RB Groningen, The Netherlands; 5 Department of Hematopathology, Hospital Clínic, Barcelona, Spain

Dear Editor,

A 70-year-old male presented with pancytopenia and massive splenomegaly. Bone marrow examination and flow cytometry showed an extensive infiltration by hairy cell leukemia variant (HCLv) with phenotypical expression of CD20, CD22, CD11c, and CD103 and lack of CD5 and CD25 (Fig. 1A–C) [1]. Cytogenetic analysis showed a complex karyotype with loss of 17p13.3p11.2. Mutational analysis by next-generation sequencing demonstrated a mutation in TP53 (c645T > A, variant allele frequency 0.44).

**Fig. 1:**
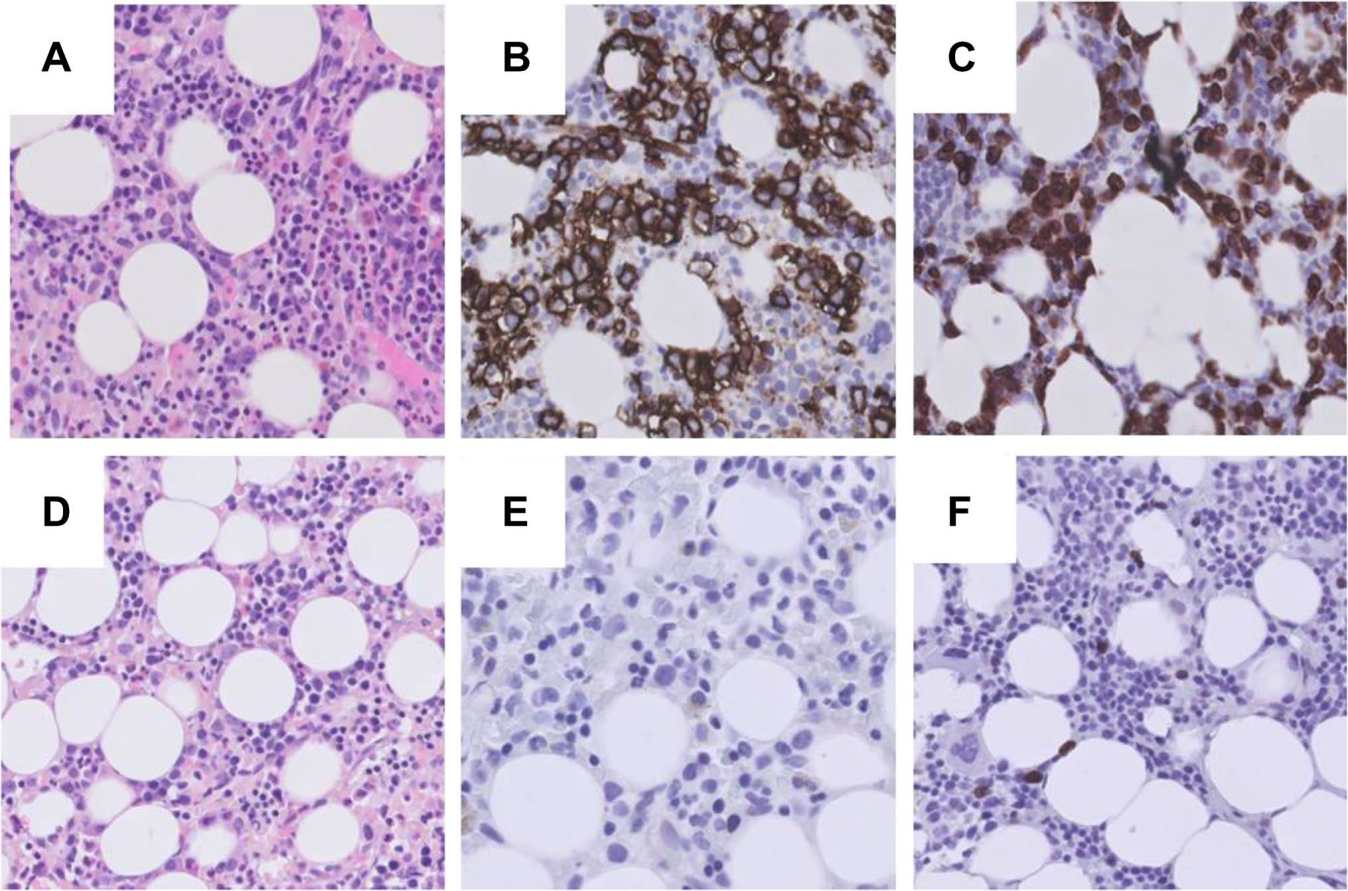
Immunohistochemical staining of bone marrow biopsy of hairy cell leukemia variant (× 400). **A** Bone marrow biopsy (H/E stain) at diagnosis showing a interstitial infiltrate of atypical lymphoid cells with enlarged, irregularly shaped nuclei. **B**, **C** Tumor cells were CD20 and CD79a positive. **D **Bone marrow biopsy (H/E stain) after successful treatment with obinutuzumab showing normal hematopoiesis. **E**, **F** No CD20- or CD79a-positive B cells could be observed. This was confirmed by flow cytometry (detection < 0.01%)

The patient was initially treated with cladribine (0.15 mg/kg/day for 5 days). Bone marrow evaluation 6 weeks after treatment showed refractory disease. The patient subsequently started rituximab at a dose of 375 mg/m^2^, which had to be discontinued after the third infusion because of an anaphylactic reaction. Rituximab plasma levels could not be detected due to anti-rituximab antibodies. Because of persistent splenomegaly, the patient subsequently underwent a splenectomy. Histological examination of the spleen showed displacement of the normal architecture by extensive diffuse infiltration of the red pulp by HCLv. Response evaluation 6 weeks after splenectomy showed persistent cytopenia due progressive HCLv bone marrow involvement. Given the absence of detectable rituximab in the plasma and the persistent CD20 expression on the tumor cells, the patient was considered naïve to CD20-targeted therapy. The fully human anti-CD20 antibody ofatumumab has been successfully used to treat rituximab-intolerant patients, but is no longer marketed in Europe [2]. Obinutuzumab is a humanized glycoengineered type 2 anti-CD20 monoclonal antibody, targeted at a different epitope of CD20 than rituximab and ofatumumab [3]. Because of these pharmacological differences between rituximab and obinutuzumab, we did not expect obinutuzumab to cause a cross hypersensitivity reaction [4]. Although obinutuzumab has not been reported in HCLv, it has been successfully employed in multidrug-resistant HCL [5].

After written informed consent, the patient received 4 weekly cycles of obinutuzumab at dosage of 1000 mg. The first infusion was given over 2 days, at a dose of 100 mg and 900 mg, respectively [3]. Infusion of obinutuzumab was uneventful. Blood counts normalized within 4 weeks after the start of the treatment. A bone marrow examination showed a complete remission with minimal residual disease (MRD) detected by flow cytometry (3% monoclonal B cells). The patient received consolidation with 4 weekly cycles of obinutuzumab. Six months after treatment, the patient remains in complete remission without detectable MRD (< 0.01% monoclonal B cells) (Fig. 1D–F).

In conclusion, this case report shows the feasibility of obinutuzumab in patients allergic to rituximab. Obinutuzumab is a potential treatment option for patients with HCLv.

## References

[1] Swerdlow SH, Campo E, Harris NL, et al. WHO classification of tumours of haematopoietic and lymphoid tissues. Lyon 2017; 230–231

[2] Chen LY, Shah R, Cwynarski K, Lambert J, McNamara C (2019). Ofatumumab is a feasible alternative anti-CD20 therapy in patients intolerant of rituximab. Br J Haematol.

[3] Goede V, Fischer K, Busch R, Engelke A, Eichhorts B (2014). Obinutuzumab plus chlorambucil in patients with CLL and coexisting condition. NEJM.

[4] Ghione P, Joffe E, De Paola N, Mainardi T, Noor SJ (2020). Alternative anti-CD20 antibody versus desensitization for lymphoma patients with drug hypersensitivity reactions requiring discontinuation of rituximab, obinutuzumab, or ofatumumab. J Clin Oncol.

[5] Bohn JP, Willenbacher E, Steurer M (2016). Obinutuzumab in multidrug-resistant hairy cell leukemia. Ann Hematol.

